# Proteomic Analysis of Human Blister Fluids Following Envenomation by Three Snake Species in India: Differential Markers for Venom Mechanisms of Action

**DOI:** 10.3390/toxins11050246

**Published:** 2019-04-30

**Authors:** Jéssica K. A. Macêdo, Joseph K. Joseph, Jaideep Menon, Teresa Escalante, Alexandra Rucavado, José María Gutiérrez, Jay W. Fox

**Affiliations:** 1 Department of Microbiology, Immunology and Cancer Biology, University of Virginia, School of Medicine, Charlottesville, VA 22908-734, USA; macedojka@gmail.com; 2 Department of Cell Biology, Brazilian Center of Protein Research, University of Brasilia, Brasilia/DF 70297-400, Brazil; 3 Little Flower Hospital, Angamaly 683572, India; drjosephkjoseph@gmail.com; 4 Sree Naryana Institute of Medical Science, Kerala 683594, India; menon7jc@gmail.com; 5 Instituto Clodomiro Picado, School of Microbiology, University of Costa Rica, San José 11501-2060, Costa Rica; teresa.escalante@ucr.ac.cr (T.E.); alexandra.rucavado@ucr.ac.cr (A.R.); jose.gutierrez@ucr.ac.cr (J.M.G.)

**Keywords:** proteomics, blister fluid, wound exudate, snake venom, inflammation, extracellular matrix, snake venom metalloproteinases (SVMP)

## Abstract

Skin blistering as a result of snakebite envenomation is characteristic of some bites, however little is known regarding the mechanism of blister formation or the composition of the blister fluid. In order to investigate if blister fluid proteomes from humans suffering snakebite envenomation could provide insights on the pathophysiology of these skin alterations, blister fluid was collected from six patients upon presentation at a clinic in India bitten by three species of snakes, *Daboia russelii* (3), *Hypnale hypnale* (2), or *Naja naja* (1). Standard clinical data were recorded throughout the treatment. Approximately 805 proteins were identified in blister fluids using proteomic analyses. Informatics analyses of the proteomes identified the top biological response categories as: platelet degranulation, innate immune response, receptor-mediated endocytosis, complement activation, and blood coagulation. Hierarchical clustering did not show a clear segregation of patients’ proteomes being associated with the species of snake involved, suggesting that either the proteomic profiles described reflect a general response to venom-induced tissue damage or more patient data sets will be required to observe significant differences. Finally, it is of interest that venom proteins were also identified in the blister fluids suggesting that this fluid may serve as a reservoir of venom biologically active proteins/toxins, and as such, may indicate the clinical value of removing blister fluid to attenuate further tissue damage.

## 1. Introduction

Snakebite envenomations represent a significant public health problem observed on a global basis, but particularly in sub-Saharan Africa, Asia, Latin America, and parts of Oceania, and have been included by the World Health Organization (WHO) in the list of neglected tropical diseases [1]. India is the country that bears the highest number of cases and it has been estimated that 50,000 people die each year in this country due to snakebites [2].

Among the many species of venomous snakes that inhabit India, four have been considered as causing the heaviest toll: the Indian cobra (*Naja naja*), the common krait (*Bungarus caeruleus*), the Russell’s viper (*Daboia russelii*), and the saw-scaled viper (*Echis carinatus*); these species comprise the group known as “the big four” [3]. In addition, it has been observed that, in certain regions, the hump-nosed pit viper species (*Hypnale hypnale*) inflicts a high number of bites and is capable of causing serious accidents, underscoring the need to further study the epidemiology of snakebites in India [4]. Although polyvalent snake antivenoms (SAV) are produced by several manufacturers in India against venoms of the “big four” species, envenomations continue to cause fatalities and pathophysiological sequelae in thousands of people a year. Thus, there is a need to further expand our knowledge on the various aspects of snakebite envenomation in this country, including a better understanding of the pathophysiology and mechanisms of action of venom-induced effects. A rapid, personalized and highly sensitive diagnosis of envenomation and identification of the venom is one of the essential needs for effective injury management. Thus, efforts have been directed at the identification of markers of tissue damage, especially at the injury site, since they may provide valuable information about the mechanisms of disease and the condition and prognosis of the patient, all of which may lead to a more effective treatment [5,6,7]

One of the characteristic manifestations of the local tissue damage inflicted by venoms of snakes from the family Viperidae, and by some species of the family Elapidae, is the formation of blisters, resulting in the accumulation of a proteinaceous fluid as a consequence of the collection of wound exudate [1]. Thereby, the study of wound exudate and blister fluids as a potential source of biomarkers have emerged as a new approach to study the effects of venoms and may assist in the diagnosis and treatment of these patients [5,8]. The proteomic analysis of fluids collected from injured tissue, and from tissue undergoing healing processes, has allowed the identification of markers that differentiate between healing and non-healing wounds, with mediators characteristic of tissue formation or mediators characteristic for a persistent inflammatory and tissue damaging response, respectively [7]. Moreover, immunochemical analysis of blister fluid from an envenomated patient has been used to identify the presence of venom components in the fluid [9].

Since viperid snake venoms induce the formation of blisters and an inflammatory exudate in the damaged tissues, a number of experimental studies have addressed the analysis of the proteomes of exudates collected from mice injected with venoms or isolated toxins. A comparative proteomic analysis of wound exudates caused by BaP1, a snake venom metalloproteinase (SVMP), and Mtx-I, a myotoxic phospholipase A2 (PLA2), showed that the SVMP causes degradation of nonfibrillar collagens, whereas PLA2-mediated tissue damage results in the presence of fibrillar collagen type I fragments; apolipoproteins A-I, A-IV, and E; and fibronectin in the exudates [5]. Moreover, the use of inhibitors demonstrated that myotoxic PLA2s and hemorrhagic SVMPs are involved in blister formation and skin damage induced by *Bothrops asper* venom [10]. Furthermore, the presence of matrix metalloproteinases (MMPs) in these exudates was observed, hence suggesting the combined action of endogenous and exogenous proteinases in these pathological events [11,12].

In a recent study, the exudates generated by SVMPs of the PI, PII, and PIII classes revealed specific proteins associated with the mechanism of action of the SVMPs [13]. Exudates from tissue injected with PI and PII SVMPs contained a higher abundance of keratins as compared to samples from mice injected with a PIII SVMP. In agreement, a PI SVMP has been shown to induce blistering in the skin of mice [14]. Hence the study of the composition of exudates and blister fluid has proven to be an information-rich approach to assess pathological processes taking place in tissues injected with snake venoms and toxins. 

Despite these advances at the experimental level, to our knowledge, there have been no attempts to investigate the composition of blister fluid or exudates in humans resulting from snakebite envenomations. The proteome analysis of fluids collected from affected tissue is therefore an experimental approach with great potential for biomarker identification by allowing the assessment of tissue alterations associated with the pathology of envenomation. In this study, utilizing mass spectrometry, we investigated the composition of blister fluid collected from patients envenomated by three species of Indian snakes, i.e., *Daboia russelii* and *Hypnale* sp. (family Viperidae), or *Naja naja* (family Elapidae).

## 2. Results and Discussion

### 2.1. Clinical Features of Envenomation

The basic demographic and clinical information of the six patients included in this study are shown in Table 1. Three of them were bitten by *D. russelii*, two by *Hypnale* sp, and one by *Naja naja*. Ages ranged from 2 to 67 years old. The time lapse between the bite and the arrival to the hospital ranged between 2 and 8.5 h. Envenomation by *D. russelii* was characterized by local manifestations (swelling, blisters), and one of them presented evidence of systemic capillary leakage syndrome (parotid swelling, conjunctival chemosis) and thrombocytopenia. Three patients developed unclottable blood, as evidenced by prolongation of the 20 min whole blood clotting test, and acute kidney injury (AKI) was present in two of them, as evidenced by elevated serum creatinine concentration (Table 1).

The two patients suffering bites by *Hypnale* sp. presented local manifestations (local pain, swelling and discoloration in the skin), and one of them indicated abdominal pain. One patient showed a prolonged 20 min whole blood clotting test, thrombocytopenia, and AKI, with elevated serum creatinine concentration (Table 1). The patient suffering from a cobra (*N. naja*) bite presented with local alterations (swelling and bluish discolouration) and manifestations of systemic neurotoxicity (ptosis and external ophtalmoplegia). 

Patients suffering envenomations by *D. russelii* and *N. naja* received 20 vials (200 mL total) of polyspecific antivenom, whereas no antivenom was administered to the patients bitten by *Hypnale* sp. since this antivenom did not include *Hypnale* sp. venom in the immunizing mixture. The patient bitten by *N. naja* was treated with polyspecific antivenom (200 mL) and neostigmine, and those who developed acute kidney injury underwent hemodialysis (Table 1). All patients survived.

### 2.2. Proteomic Analysis of Blisters

**Figure 1:**
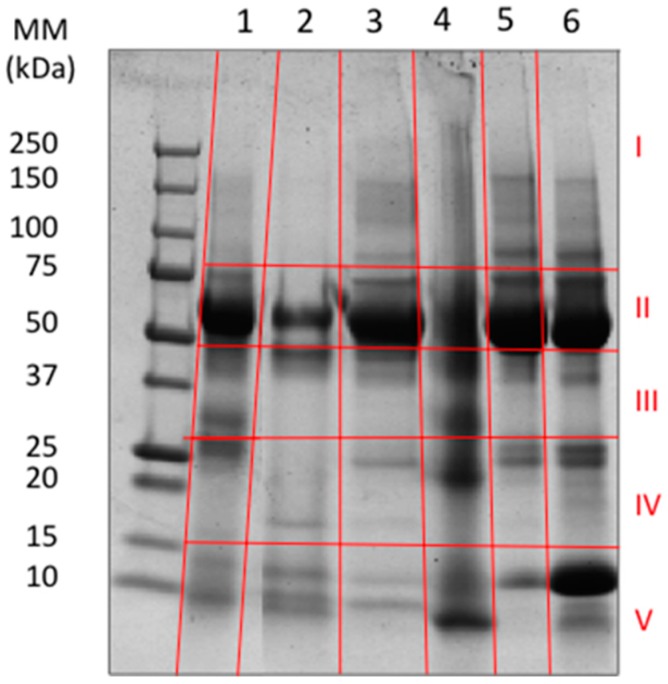
SDS-PAGE profile of samples of blister fluid collected from patients. Samples were run on a 12% gel and stained with Coomassie brilliant blue. Samples from patients bitten by *N. naja* (lane 1); *Hypnale* sp. (lanes 2 and 4); *D. russelii* (lanes 3, 5, and 6). The left lane corresponds to molecular mass markers. The red lines indicate the locations in which the bands were cut for analysis using LC-MS/MS.

Blister fluid obtained from these patients were first analyzed using SDS-PAGE gel electrophoresis. For some samples, there appeared to be a correlation between the gel profiles and the species of snakes causing the bites. For others, however, there were differences even when comparing blisters obtained from patients bitten by the same species. This was observed in the case of the two patients bitten by *Hypnale* sp. (Figure 1). After electrophoresis, bands digestion, and LC-MS assay, the proteomic data were obtained and various analyses were performed. Mass spectrometry results identified a total of 805 different proteins in blisters collected from the patients (Supplementary Table S1) Many of the proteins detected in this analysis have also been described in laboratory studies collecting exudate fluid from the tissue of mice injected with the venom of the Central American pit viper *Bothrops asper* or with purified hemorrhagic SVMPs or myotoxic PLA_2_s isolated from this and other viperid venoms [5,13,15]. The individual protein identification was considered significant with a minimum of two peptides detected and protein identification probability above 95%. 

**Figure 2:**
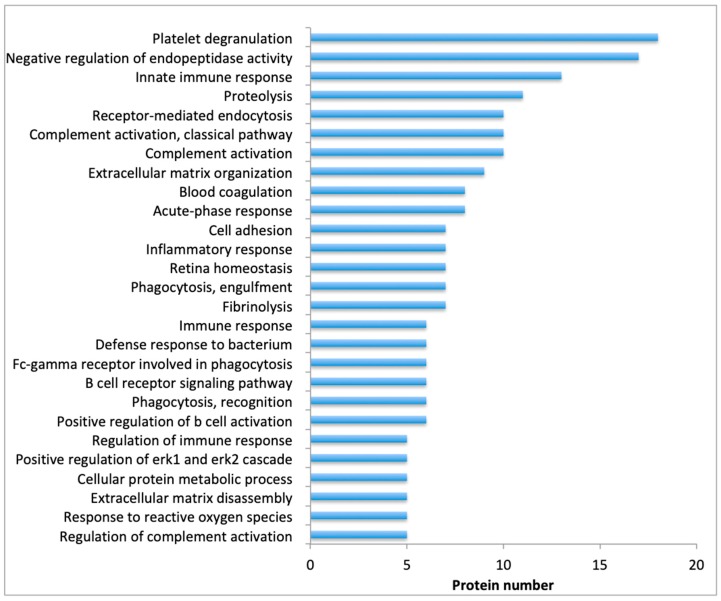
Top biological responses, identified via DAVID bionformatic analysis, of the proteomes of blisters’ samples collected from the patients [16,17]. The number of proteins detected corresponding to each process is indicated.

The most abundant proteins based on their quantitative value are presented in Table 2 and the 15 most abundant proteins in each exudate are highlighted in gray. Informatics analysis of the proteomes identified the top biological response categories as: platelet degranulation, negative regulation of endopeptidase activity, innate immune response, proteolysis, receptor-mediated endocytosis, complement activation, extracellular matrix organization, and blood coagulation (Figure 2). Such events illustrate the local and systemic events triggered in the course of envenomation in terms of change in metabolism, secretion, and synthesis of diverse components as a response to the action of snake venom in the tissues.

Proteins were classified in groups according to functional characteristics, using Gene Ontology within the DAVID Gene Functional Classification Tool (http://david.abcc.ncifcrf.gov). From the pathophysiological standpoint, it was of interest to analyze proteins of the coagulation system (Table 3) and from the extracellular matrix (ECM) (Table 4), as coagulopathies and ECM degradation are characteristic of viperid snakebite envenomation. Regarding proteins of the coagulation system, fibrinogen and plasminogen were the most abundant, with differences noted between patients. Blister fluid from patient 3 (bitten by *D. russelii*), who showed the most severe clinical manifestations of envenomation (Table 1), presented a higher abundance for fibrinogen alpha-chain and plasminogen. Interestingly, the sample from the patient bitten by the cobra *N. naja*, also contained fibrinogen, although cobra venoms do not cause significant effects on coagulation. This finding is probably a consequence of plasma extravasation following cobra venom-induced local tissue damage.

The analysis of extracellular matrix (ECM) proteins provided interesting findings (Table 4). Samples from patients 3 (*D. russelii*) and 4 (*Hypnale* sp.) presented fragments of heparan sulphate proteoglycan core protein and type IV collagen, two key components of basement membranes. Hydrolysis of these proteins has been associated, in experimental studies in mice, with the action of hemorrhagic SVMPs and with the pathogenesis of microvascular damage leading to extravasation [13,18]. The presence of these proteins in blister fluid is indicative of basement membrane degradation, possibly associated with local hemorrhage and blistering. Again, in the case of patient 3 bitten by *D. russelii*, these findings correlated with the severe manifestations of envenoming. In this patient, tenascin-X and subunit alpha-5 of laminin were also detected in the blister fluid, further corroborating the ECM damage induced by *D. russelii* venom. Fragments of tenascin-X were also found in exudates collected from mice injected with hemorrhagic SVMPs [5,13,18]. In addition, blister fluid from patients 4 (*Hypnale* sp.) and 1 (*Naja naja*) contained fragments of type VI collagen. Experimental studies in mice injected with SVMPs have shown similar findings in exudates collected from damaged muscle tissue [13,18]. Type VI collagen is known to connect the basement membrane with fibrillar collagens, hence integrating the basement membrane with the surrounding matrix. Hydrolysis of this collagen may also have implications for the pathogenesis of local tissue damage. Our findings underscore the value of the proteomic analysis of blister fluid to assess ECM degradation in snakebite envenoming. 

### 2.3. Protein Clustering

**Figure 3:**
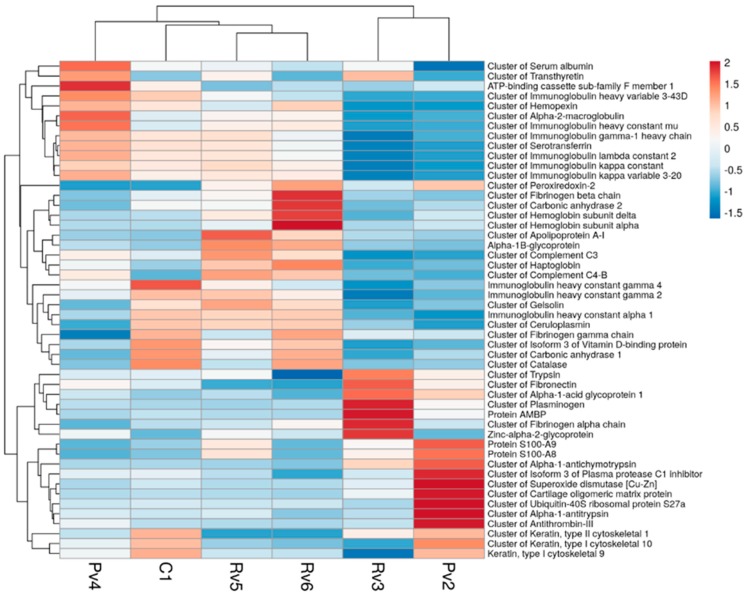
Hierarchical clusters created from the proteomic profile of blisters’ samples collected from the patients. Analysis was performed using the Clutvis software [19]. The colors represent a scale on the level of expression that goes from red, representing high abundance proteins, to blue, representing low abundant proteins. Pit viper (Pv) corresponds to *Hypnale* sp., Rv corresponds to *Daboia russelii*, and C corresponds to *Naja naja.*

Hierarchical clustering created from the proteomic profile did not show a clear segregation on the basis of the species of snake causing envenomation (Figure 3). This may have a number of possible explanations including: (a) The set of proteins present in blisters is mostly a consequence of tissue damage and the inflammatory response to tissue pathology, regardless of the venom responsible for the damage. The three species involved in this study are known to induce local tissue damage. (b) The set of proteins found is highly dependent on the severity of the damage and not on the species causing the bite. (c) The number of patient blisters analyzed by a particular snake was insufficient to power a significant observation of differences in response. Lastly, (d) the proteomics of blister fluid likely is also time dependent and thus these samples collected at different times might mask differences due to the species of snake. As indicated in Table 1, there was a wide variation in the severity of envenomation within the patients studied. In agreement, patient 3, who suffered a severe envenomation from *D. russelii*, presented the highest abundance of some clotting proteins and of ECM proteins, in contrast with the other two patients affected by this species. Whatever the case, it is necessary to study the proteomics of blister fluid of a larger number of patients showing a spectrum of severity and at different times after the bite in order to have a comprehensive view of the changes in the proteomic profile of blister fluid in different circumstances of envenomation. 

### 2.4. Identification of Proteins from the Snake Venoms in Blisters

**Figure 4:**
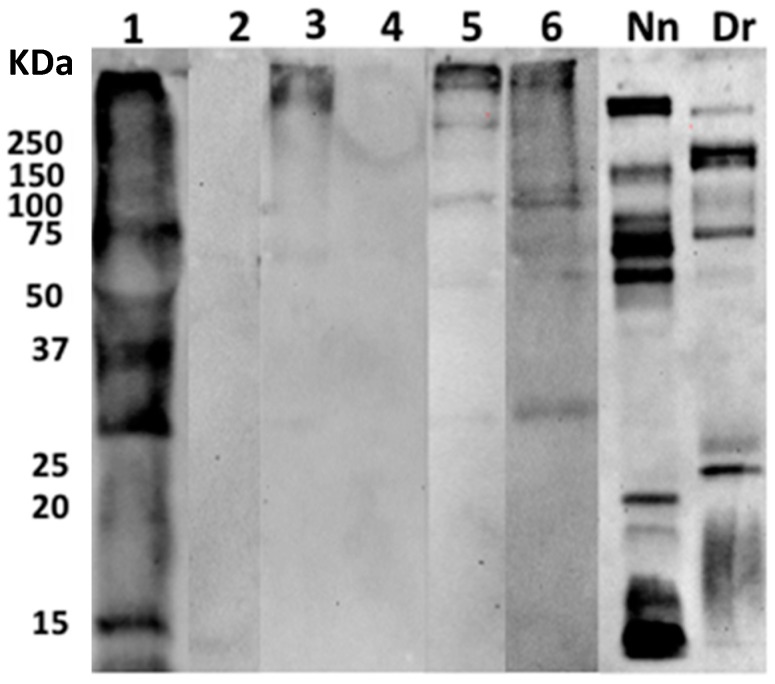
Detection of venom proteins in human blisters using Western blotting. Blister samples were separated under non-reducing conditions on 12% SDS-PAGE gels and were transferred to a nitrocellulose membrane. Immunodetection was performed with polyspecific VINS antivenom. The reaction was detected using an anti-horse peroxidase antibody and a chemiluminescent substrate. Lanes 1 to 6 correspond to samples of blister fluid (see Table 1). Nn: *N. naja* venom, Dr: *D. russelii* venom run in parallel to the blister samples.

Venom proteins were identified in the blister fluids using proteomics and Western blotting. Using proteomics, venom proteins of various families were identified in two patients bitten by *D. russelii* and *N. naja* (Table 5). In the case of patient 3 (*D. russelii*), this is in agreement with the clinical manifestations that showed a high severity envenomation (Table 1). On the other hand, detection of venom proteins using immunoblotting performed with Indian polyspecific antivenom was able to identify snake venom proteins in several samples. The three Russell’s viper samples exhibited proteins imaged by Western blot in the high molecular mass range, plus some between 40 and 75 kDa and another of around 25 kDa. The sample from the *N. naja* case, in turn, presented not only a greater intensity but also a higher number of bands (Figure 4). Blister fluid from the patient bitten by *N. naja* was therefore the sample with a higher abundance of venom detected using both mass spectrometry and Western blot analyses. The abundance of venom proteins present in the blister fluid in the case of the patient bitten by *N. naja* may be related to the fact that cobras usually inject their venoms subcutaneously, whereas viperid venoms generally inject venom deeper in the tissues. The lower amount of proteins identified by proteomics in some samples as compared to Western blot may be due to the scarcity of venom sequence databases for mass spectrometry analysis, which has been a challenge for all venom proteomic studies. On the other hand, the low reactivity of some samples in the Western blot might be due to variations in the immunoreactivity of antivenom antibodies against some venom components. Immunochemical detection of venom of the Taiwanese snake *Protobothrops mucrosquamatus* was described in the blister fluid collected from a patient [9]. 

## 3. Conclusions

Previous studies carried out in mice have described the potential of proteomics analysis of wound exudate fluid to provide a “window” to explore the pathological and inflammatory events occurring in tissues injected with snake venoms. The present investigation expanded this type of analysis to the fluid of blisters present in human patients proximal to the anatomical site of venom injection in bites inflicted by three different snake species present in India. The results corroborate the value of proteomics analysis for detecting a variety of tissue plasma and inflammatory proteins of different origin as a consequence of the tissue-damaging action of these venoms and the subsequent tissue response through inflammation and repair. Although some differences were observed between samples from patients bitten by different species, overall there was no clear segregation on the basis of the offending species via hierarchical clustering analysis. Therefore, it would be of value in the future to expand these analyses to a larger group of patients in order to assess whether specific tissue markers may characterize different types of envenomation, and whether different proteomic patterns occur depending on the severity and time-course of envenomation. Notably, venom was detected in the blister fluid, as observed by both mass spectrometric and immunochemical analyses. Overall, our findings underscore the use of proteomics analysis of exudate and blister fluids of patients suffering snakebite envenomations for further advancing the understanding of the pathophysiology of this neglected tropical disease. Furthermore, the presence of venom in blister fluid suggests that it may represent a reservoir of venom for further diffusion into the tissues, hence supporting the concept that aseptic removal of blister fluid may reduce the extent and time-dependence of venom-induced tissue damage.

## 4. Materials and Methods

### 4.1. Clinical Evaluation and Treatment

Upon admission to hospital, routine clinical and laboratory evaluations were carried out for all patients. The hospital’s routine treatment protocols were followed, including, when appropriate, the administration of polyspecific Indian antivenom, and various interventions that depended on the clinical evolution of each case. Identification of the offending snake was done via direct examination of the specimen, brought in by the patient or his/her relatives.

### 4.2. Sample Collection

Fluid was aspirated from the blister under sterile conditions using a syringe. Two to five milliliters of volume was typically collected from a single blister.

### 4.3. Ethics Statement

The Ethical Committee of the Little Flower Hospital and Research Centre, Algamaly, India, authorized the project (“Blister fluid of snake bite victims analysis,” IRB session held on May 6, 2012). An informed consent was obtained from participating patients.

### 4.4. Proteomic Analysis of the Exudates

#### 4.4.1. SDS-PAGE

Lyophilized blister samples were kept frozen (−18 °C) until time of use. Protein quantification was performed using a micro BCA protein assay kit (Thermo Scientific, Waltham, WA, USA),. Twenty micrograms of protein were further resuspended in Laemmli buffer, applied to a 12% precast electrophoresis gel (Bio-Rad, Hercules, CA, USA), separated, and stained with 0.1% Coomassie Brilliant Blue in 40% methanol, 10% acetic acid for about 1.5 h, then distained with 40% methanol, 10% acetic acid, then in 5% acetic acid.

#### 4.4.2. Protein Extraction and Digestion

Gel lanes were cut in ten equal-sized slices, which were destained for 3 h, and the proteins reduced with 10 mM dithiothreitol (DTT) and alkylated with 50 mM iodoacetamide at room temperature. Gel slices were washed with 100mM ammonium bicarbonate, dehydrated with acetonitrile, and dried in a speed vac. A solution of Promega modified trypsin (20 ng/μL) in 50mM ammonium bicarbonate was used to rehydratate the samples for 30 min on ice. Excess trypsin solution was removed and the digestion was carried on for an additional 18 h at 37 °C. Tryptic peptides were twice extracted from gel slices with 30 μL of a 50% acetonitrile/5% formic acid solution. The extracts were dried to a volume of 15 μL for mass spectrometric analysis.

#### 4.4.3. Proteomic Analysis

A Thermo Electron Orbitrap Velos ETD mass spectrometer system was used to perform the LC/MS/MS experiments. Analytical columns were made by packing 0.5 cm of irregular C18 Beads (YMC Gel ODS-A, 12 nm, I-10-25 μm) followed by 7.5 cm Jupiter 10 μm C18 packing material (Phenomenex, Torrance, CA, USA) into 360 × 75 μm fused silica (Polymicro Technologies, Phoenix, AZ, USA) behind a bottleneck. Aliquots of 7 μL were loaded directly onto these columns and then eluted into the mass spectrometer at 0.5 μL/min using a 1h gradient consisting of acetonitrile/0.1M acetic acid (2–90% acetonitrile). The instrument was set to a full MS (m/z 300–1600) resolution of 60,000 and programmed to acquire a cycle of one mass spectrum followed by collision-induced dissociation (CID) MS/MS performed in the ion trap on the twenty most abundant ions in a data-dependent mode. Dynamic exclusion was enabled with an exclusion list of 400 masses, duration of 60 seconds, and repeat count of 1. The electrospray voltage was set to 2.4 kV, and the capillary temperature was 265 °C. Peak lists were generated from the raw data against the Uniprot Human and NR database from July 2014 using the Sequest search algorithm in Proteome Discoverer 1.4.1. Analysis of the spectra generated was performed using carbamidomethylation on cysteine as a fixed modification, oxidation of methionine as a variable modification, 10 ppm parent tolerance, and 1 Da fragment tolerance. All hits were required to be fully tryptic. 

For analysis of the results and validation of peptide and protein identifications, data obtained were exported to Scaffold (version 4.3.2, Proteome Software Inc., Portland, OR, USA). Protein identifications were filtered using Xcorr cutoff values dependent on charge state (+1 >1.8, +2 >2.2, +3 >2.5, and +4 >3.5). Relative quantization of proteins was accomplished by grouping all data from the 10 gel slices for a particular sample in Scaffold and then displaying the quantitative value. This number gives an average total of non-grouped spectral counts for a protein divided by the total non-grouping spectral counts for the 10 mass spectral runs from the gels slices from each lane (http://www.proteomesoftware.com/) and allows a relative quantitative comparison between specific protein from different samples.

Hierarchical clustering analysis was performed using ClustVis (http://biit.cs.ut.ee/clustvis/) to determine any clusters of samples or proteins. To create heat maps, the list of proteins obtained using proteomic analysis was log transformed and both rows and columns were clustered using Euclidean distance and average linkage. DAVID (https://david.ncifcrf.gov/) was applied to the list of the most abundant proteins in order to identify gene ontology terms and KEGG pathways or over-represented biological processes.

### 4.5. Western Blot

Detection of venom proteins in human wound exudate was done using Western blotting. Exudate samples were separated under non-reducing conditions on 12% SDS-PAGE gels and were transferred to a nitrocellulose membrane. Immunodetection was performed with polyvalent VINS polyspecific antivenom (VINS Bioproducts Limited, India, batch No. 01AS13100). The reaction was detected using an anti-horse peroxidase antibody and a chemiluminiscent substrate. This antivenom is an F(ab’)_2_ preparation, produced from the plasma of horses immunized with venoms of the Indian species *Naja naja*, *Bungarus caeruleus, Daboia russelii*, and *Echis carinatus*.

## Figures and Tables

**Table 1:** Clinical and laboratory parameters of the six patients whose blister fluid was analyzed in this work.

Snake Species	*Naja naja*	*Hypnale* sp.	*Daboia russelii*	*Hypnale* sp.	*Daboia russelii*	*Daboia russelii*
Patient number	1	2	3	4	5	6
Sex	Male	Male	Male	Female	Male	Female
Age	53 years old	62 years old	22 years old	2 years old	23 years old	67 years old
Bite site	Left thumb	Left big toe	Left leg	Left index finger	Left ankle	Right leg
Time between the bite and arrival in the hospital	2 h	4 h 20 min	8 h	4 h 15 min	8 h 35 min	3 h 20 min
Symptoms	Ptosis and external ophthalmoplegia developed 1 h after arrival to the hospital. Local pain, swelling, and bluish discoloration of left thumb. No nausea, vomiting nor abdominal pain.	Local pain, swelling, and bluish discoloration of left big toe. Nausea, vomiting, and abdominal pain present.	Disorientation, left leg swelling, and blisters. Under the influence of alcohol at the time of bite. Bilateral parotid swelling and conjunctival chemosis. Nausea, vomiting, and pain	Local pain, swelling, and discoloration and blister on the left index finger. No nausea, vomiting, nor abdominal pain.	Local pain and swelling. Proteinuria. Nausea, vomiting, and abdominal pain present.	Local pain and swelling Bite mark present. Nausea and vomiting present, and no abdominal pain present.
Platelet count	177,000/µL	70,000/µL	20,000/µL	300,000/µL	160,000/µL	120,000/µL
Hemoglobin	12.8 g/dL	10 g/dL	15.7 g/dL	11.7 g/dL	17.9 g/dL	14.1 g/dL
Serum creatinine	0.86 mg/dL	3.47 mg/dL	7.94 mg/dL	0.41 mg/dL	2.40 mg/dL	0.80 mg/dL
Serum K^+^	4.1 mEq/L	3 mEq/L	4.8 mEq/Ll	4 mEq/L	6.7 mEq/L	
Serum Na^+^		126 mEq/L	133 mEq/L	130 mEq/L	131 mEq/L	
Whole blood clotting time	Not determined	>20 min	>20 min	12 min	>20 min D dimer 4 µg/mL (positive) (normal range <0.5 µg/mL)	>20 min
Treatment	20 vials antivenom, with neostigmine given 1 h after antivenom; ophthalmoplegia and ptosis disappeared.	No antivenom was administered. Patient had acute kidney injury, underwent hemodialysis.	20 vials of antivenom. Patient had acute kidney injury, underwent hemodialysis.	Patient treated symptomatically. No antivenom was administered.	20 vials of antivenom. Patient had acute kidney injury, underwent hemodialysis.	20 vials of antivenom. Early adverse reaction to antivenom that was managed with adrenaline.
Observations	Multiple tourniquets applied	No tourniquet was applied	No tourniquet was applied	Tourniquet applied	Traditional medicine received prior to reaching the hospital. Tourniquet applied.	No tourniquet was applied

**Table 2:** The most abundant proteins identified in each blister’s samples via proteomic analysis. The top 15 proteins by sample are highlighted in gray. Proteins were identified from the raw data using the Sequest search algorithm Proteome Discovery 1.4.1 against a nonspecific database and the functional annotation provided by DAVID Bioinformatics Resources 6.7.

Identified Proteins	Accession	*D.russelli*			*Hypnale* sp.		*N.naja*
		3	5	6	2	4	1
Cluster of serum albumin	P02768	1892	1837	1620	1142	2525	1891
Cluster of alpha-1-antitrypsin	P01009	85	139	37	625	136	102
Cluster of serotransferrin	P02787	1	349	267	46	443	366
Cluster of hemoglobin subunit delta	P02042	0	341	664	144	86	105
Cluster of keratin, type II cytoskeletal 1	P04264	272	64	65	364	155	371
Cluster of immunoglobulin kappa constant	P01834	25	345	282	64	359	306
Cluster of complement C3	P01024	0	355	271	18	220	148
Cluster of hemoglobin subunit alpha	P69905	43	221	891	84	46	55
Cluster of alpha-1-antichymotrypsin	P01011	349	52	20	521	58	58
Cluster of alpha-2-macroglobulin	P01023	1	177	182	24	345	126
Cluster of immunoglobulin gamma-1 heavy chain	P0DOX5	29	262	190	81	301	249
Cluster of trypsin	P00761	140	100	44	106	88	91
Cluster of immunoglobulin lambda constant 2	P0DOY2	12	151	124	29	193	149
Cluster of carbonic anhydrase 1	P00915	0	108	230	54	22	249
Cluster of keratin, type I cytoskeletal 10	P13645	0	39	24	243	104	205
Cluster of transthyretin	P02766	96	77	44	41	103	49
Cluster of haptoglobin	Q62558	4	150	186	17	86	31
Cluster of alpha-1-acid glycoprotein 1	P02763	203	59	27	153	65	45
Cluster of antithrombin-III	P01008	34	29	25	209	67	31
Cluster of isoform 3 of plasma protease C1 inhibitor	P05155-3	37	30	10	119	45	46
Cluster of complement C4-B	P0C0L5	23	58	53	21	45	22
Keratin, type I cytoskeletal 9	P35527	0	35	33	83	52	85
Cluster of hemopexin	P02790	12	39	73	13	80	65
Cluster of ubiquitin-40S ribosomal protein S27a	P68202	0	8	11	139	8	16
Cluster of peroxiredoxin-2	P32119	26	50	81	71	0	1
Cluster of Immunoglobulin heavy constant mu	P01871	0	42	45	3	76	25
Cluster of fibronectin	P02751	159	7	2	85	78	52
Protein S100-A9	P06702	45	55	6	96	5	14
Cluster of fibrinogen gamma chain	P02679	29	24	55	25	5	53
Cluster of fibrinogen beta chain	P02675	5	34	90	0	0	23
Immunoglobulin heavy constant alpha 1	P01876	12	70	73	3	24	74
Cluster of fibrinogen alpha chain	P02671	114	22	48	21	2	18
Cluster of isoform 3 of vitamin D-binding protein	P02774-3	0	32	48	5	13	57
Immunoglobulin heavy constant gamma 2	P01859	0	131	101	30	76	136
Cluster of superoxide dismutase	P00441	27	1	1	158	0	14
Protein S100-A8	P05109	32	39	4	61	2	6
Cluster of ceruloplasmin	P00450	10	40	41	0	2	42
Cluster of catalase	P04040	0	11	73	2	0	80
Cluster of gelsolin	P06396	0	37	30	6	4	28
Immunoglobulin constant gamma 4	P01861	8	80	50	31	70	140
Cluster of plasminogen	P00747	234	1	8	77	12	4
Cluster of apolipoprotein A-I	G3QY98	3	41	27	1	1	0
Protein AMBP	P02760	206	8	3	59	3	12
Zinc-alpha-2-glycoprotein	P25311	54	26	17	9	25	9
Cluster of immunoglobulin kappa variable 3-20	P01619	0	23	22	3	30	21
Cluster of carbonic anhydrase 2	P00918	0	10	27	3	0	10
Alpha-1B-glycoprotein	P04217	3	23	21	2	5	3
Cluster of immunoglobulin heavy variable 3-43D	P0DP04	0	24	12	1	49	40
ATP-binding cassette sub-family F member 1	Q8NE71	4	3	5	6	22	11
Cluster of cartilage oligomeric matrix protein	P49747	22	3	0	86	3	4

**Table 3:** Proteins in blister fluid in relation to coagulation. Proteins were identified from the raw data using the Sequest search algorithm Proteome Discovery 1.4.1 against the Swiss-Prot database.

Coagulation Proteins	Accession	MW	*D.russelii*			*Hypnale* sp.		*N.naja*
			3	5	6	2	4	1
Cluster of fibrinogen alpha chain	P02671	95 kDa	114	22	48	21	2	18
Cluster of fibrinogen beta chain	P02675	56 kDa	5	34	90	0	0	23
Cluster of fibrinogen gamma chain	P02679	52 kDa	29	24	55	25	5	53
Cluster of plasminogen	P00747	91 kDa	234	1	8	77	12	4
Cluster of prothrombin	P00734	70 kDa	4	0	0	0	0	0
Coagulation factor XII	P00748	68 kDa	8	0	0	0	0	0
Coagulation factor XIII B chain	P05160	76 kDa	0	0	0	1	0	0
Factor XIIa inhibitor	P50448	52 kDa	0	0	0	1	0	0
Heparin cofactor 2	P05546	57 kDa	0	4	2	0	1	0
Isoform LMW of kininogen-1	P01042-2	48 kDa	66	0	0	0	0	0
Kininogen-1	O08677	73 kDa	1	0	0	0	0	0
von Willebrand factor	P04275	309 kDa	1	0	0	7	0	0

**Table 4:** Proteins in blister fluid related to the extracellular matrix. Proteins were identified from the raw data using the Sequest search algorithm Proteome Discovery 1.4.1 against the human basic database.

Extracellular Matrix Proteins	Accession	MW	*D.russelii*			*Hypnale* sp.		*N.naja*
			3	5	6	2	4	1
Basement membrane-specific heparan sulfate proteoglycan core protein	P98160	469 kDa	66	0	0	22	0	5
Cluster of aggrecan core protein	P16112	261 kDa	1	0	0	0	0	0
Cluster of cartilage oligomeric matrix protein	P49747	83 kDa	22	0	0	0	0	0
Cluster of laminin subunit alpha-3	Q16787	367 kDa	4	0	0	0	0	0
Cluster of laminin subunit gamma-2	Q13753	131 kDa	8	0	0	0	0	0
Cluster of nidogen-2	Q14112	151 kDa	7	0	0	0	0	0
Cluster of thrombospondin-1	P07996	129 kDa	0	2	1	2	0	1
Collagen alpha-1(I) chain	P02452	139 kDa	1	0	0	6	0	0
Collagen alpha-1(I) chain (fragments)	C0HJN3	88 kDa	0	0	0	1	0	0
Collagen alpha-1(III) chain	P04258	94 kDa	0	0	0	5	0	0
Collagen alpha-1(XIV) chain	Q05707	194 kDa	21	0	0	0	0	0
Collagen alpha-1(XVI) chain	Q07092	158 kDa	0	0	0	2	0	0
Collagen alpha-1(XXVIII) chain	Q2UY09	117 kDa	1	0	0	0	0	0
Collagen alpha-2(IV) chain	P08572	168 kDa	34	0	0	17	0	1
Collagen alpha-3(VI) chain	P12111	344 kDa	3	0	0	23	0	23
Isoform 5 of tenascin-X	P22105-4	459 kDa	56	0	0	0	0	0
Isoform C of proteoglycan 4	Q92954-3	141 kDa	8	0	0	0	0	0
Laminin subunit alpha-4	P97927	202 kDa	8	0	0	1	0	0
Laminin subunit alpha-5	O15230	400 kDa	32	0	0	1	0	0
Lumican	P51884	38 kDa	0	3	0	0	0	0
Matrix metalloproteinase-9	P14780	78 kDa	0	3	0	0	0	0
Nidogen-1	P14543	136 kDa	3	0	0	6	1	0
Tenascin	P24821	241 kDa	0	0	0	1	0	0
Thrombospondin-4	P49744	108 kDa	0	0	0	2	0	0

**Table 5:** Proteins from snake venoms identified in blister fluids using proteomic analysis. Proteins were identified from the raw data using the Sequest search algorithm Proteome Discovery 1.4.1 against a nonspecific database.

Venom Proteins	Accession	MW	*D.russelii*			*Hypnale* sp.		*N.naja*
			3	5	6	2	4	1
Acidic phospholipase A2 inhibitor chain HPD-1	A4VBF0	15 kDa	3	0	0	0	0	0
Alpha-cobratoxin	P01391	8 kDa	0	0	0	0	0	16
Beta-fibrinogenase	E0Y419	28 kDa	1	0	0	0	0	0
Cluster of acidic phospholipase A2	P15445	13 kDa	0	1	0	0	0	75
Cluster of acidic phospholipase A2 Drk-a1	A8CG86	15 kDa	27	0	0	0	0	0
Cluster of acidic phospholipase A2 Tbo-E6	Q2YHJ3	16 kDa	0	0	0	2	0	46
Cluster of cytotoxin 2a	Q9PST4	9 kDa	0	0	0	0	0	46
Cluster of factor V activator RVV-V alpha	P18964	26 kDa	14	0	0	0	0	0
Cluster of Kunitz-type serine protease inhibitor C3	A8Y7N6	9 kDa	10	0	0	0	0	3
Cysteine-rich venom protein kaouthin-1	P84805	27 kDa	0	0	0	0	0	10
Hemorrhagic metalloproteinase-disintegrin-like kaouthiagin	P82942	44 kDa	0	0	0	0	0	3
Kunitz-type serine protease inhibitor 2	Q2ES49	10 kDa	48	0	0	0	0	0
Kunitz-type serine protease inhibitor 2	P00990	7 kDa	14	0	0	0	0	0
Kunitz-type serine protease inhibitor C6	A8Y7N9	10 kDa	4	0	0	0	0	0
Kunitz-type serine protease inhibitor DrKIn-I	H6VC05	10 kDa	8	0	0	0	0	6
Long neurotoxin 1	P25668	8 kDa	0	0	0	0	0	19
Muscarinic toxin-like protein 2	P82463	7 kDa	0	0	0	0	0	2
Muscarinic toxin-like protein 3	P82464	8 kDa	0	0	0	0	0	6
Neutral phospholipase A2 RVV-PFIIc’ (fragment)	P0DKX1	2 kDa	10	0	0	0	0	0
Phospholipase A2 1 (fragment)	P86529	2 kDa	3	0	0	0	0	0
Serine protease VLSP-3	E0Y420	28 kDa	8	0	0	0	0	0
Snake venom vascular endothelial growth factor toxin VR-1’	P0DL42	13 kDa	10	0	0	0	0	0
Thrombin-like enzyme 1	A7LAC6	29 kDa	3	0	0	0	0	0
Venom nerve growth factor	P30894	13 kDa	23	0	0	0	0	0
Venom nerve growth factor 2	Q5YF89	27 kDa	0	0	0	0	0	11
Zinc metalloproteinase-disintegrin-like daborhagin-K	B8K1W0	70 kDa	1	0	0	0	0	0

## Supplementary Materials

The following are available online at https://www.mdpi.com/2072-6651/11/5/246/s1, Table S1: Total proteins identified by proteomic analysis. Proteins were identified from the raw data using the Sequest search algorithm Proteome Discovery 1.4.1 against a non-specific database and the functional annotation provided using DAVID Bioinformatics Resources 6.7.
